# Ultrasound-Enhanced Ionotropic Gelation of Pectin for Lemon Essential Oil Encapsulation: Morphological Characterization and Application in Fresh-Cut Apple Preservation

**DOI:** 10.3390/foods14111968

**Published:** 2025-05-31

**Authors:** Rofia Djerri, Salah Merniz, Maria D’Elia, Nadjwa Aissani, Aicha Khemili, Mohamed Abou Mustapha, Luca Rastrelli, Louiza Himed

**Affiliations:** 1 The Biotechnology and Food Quality Research Laboratory (BIOQUAL), INATAA, University of the Brothers Mentouri-Constantine 1, Constantine 25017, Algeria; rofia.djerri@student.umc.edu.dz (R.D.); louiza.himed@umc.edu.dz (L.H.); 2 Institute of Industrial Hygiene and Safety, University Batna 2, Batna 05078, Algeria; 3 National Biodiversity Future Center (NBFC), 90133 Palermo, Italy; mdelia@unisa.it; 4 Department of Pharmacy, University of Salerno, 84084 Fisciano, Italy; 5 Dipartimento di Scienze della Terra e del Mare, University of Palermo, 90133 Palermo, Italy; 6 Faculty of Life and Natural Sciences, Department of Molecular and Cellular Biology, Water, Environment and Health, Abbes Laghrour University, Khenchela 40000, Algeria; aissani1995najma@gmail.com (N.A.);; 7 Centre de Recherche Scientifique et Technique en Analyses Physico-chimiques CRAPC, BP 384, Bou-Ismail, Tipaza 42004, Algeria; moho37@hotmail.fr

**Keywords:** lemon essential oil, pectin microcapsules, ultrasound-assisted encapsulation, fresh-cut apple preservation, antioxidant and antifungal activity

## Abstract

The growing demand for natural preservatives in the food industry has highlighted the importance of essential oils (EOs), despite their limitations related to volatility and oxidative instability. This study addresses these challenges by developing pectin-based microcapsules for encapsulating lemon essential oil (LEO) using ultrasound-assisted ionotropic gelation. The EO, extracted from Citrus limon (Eureka variety), exhibited a high limonene content (56.18%) and demonstrated significant antioxidant (DPPH IC50: 28.43 ± 0.14 µg/mL; ABTS IC50: 35.01 ± 0.11 µg/mL) and antifungal activities, particularly against A. niger and Botrytis spp. Encapsulation efficiency improved to 82.3% with ultrasound pretreatment, and SEM imaging confirmed spherical, uniform capsules. When applied to fresh-cut apples, LEO-loaded capsules significantly reduced browning (browning score: 1.2 ± 0.3 vs. 2.8 ± 0.2 in control), microbial load (4.9 ± 0.2 vs. 6.5 ± 0.4 log CFU/g), and weight loss (4.2% vs. 6.4%) after 10 days of storage at 4 °C. These results underscore the potential of ultrasound-enhanced pectin encapsulation for improving EO stability and efficacy in food preservation systems.

## 1. Introduction

**Figure 1:**
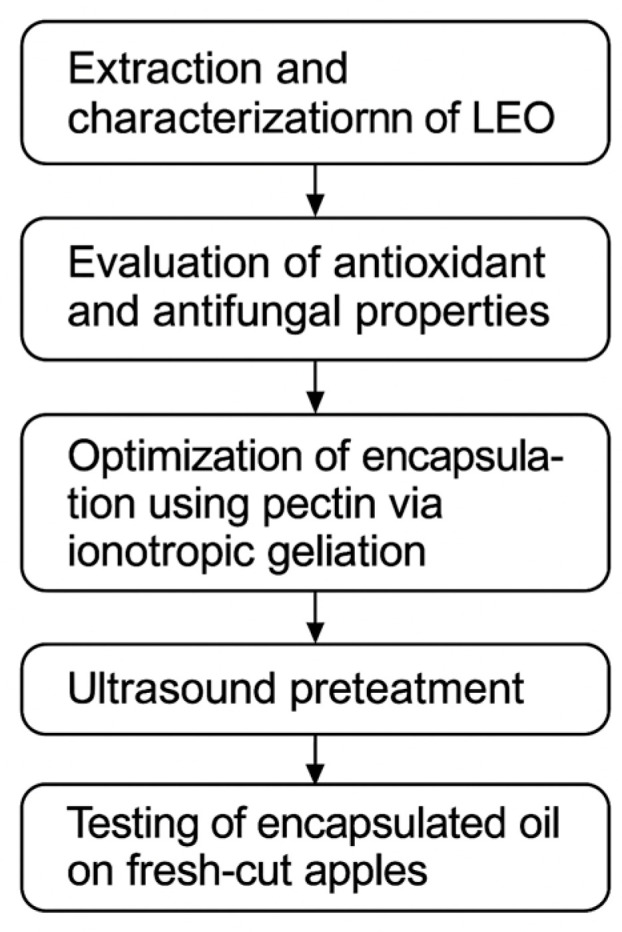
Schematic representation of the experimental protocol used in this study. The flowchart outlines the main steps: (1) extraction and characterization of lemon essential oil (LEO), (2) evaluation of its antioxidant and antifungal properties, (3) encapsulation via ionotropic gelation using pectin, (4) application of ultrasound pretreatment, and (5) testing of the encapsulated oil on fresh-cut apple slices under refrigerated conditions.

Citrus fruits, particularly lemon (Citrus limon), are widely recognized for their rich content of bioactive compounds, including flavonoids, ascorbic acid, and essential oils. Among citrus by-products, lemon peels are especially rich in volatile compounds such as limonene, which impart antioxidant, antimicrobial, and antifungal properties. These attributes make lemon essential oil (LEO) a promising candidate for application in food preservation, pharmaceuticals, and cosmetics [1]. However, the practical use of EOs is often limited by their volatility, poor water solubility, and sensitivity to light, heat, and oxygen [2]. To overcome these challenges, encapsulation technologies have been developed to protect EOs and ensure their controlled release. Natural biopolymers like pectin offer distinct advantages due to their biocompatibility, gel-forming ability, and regulatory approval for food use [3]. In particular, pectin-based matrices have been investigated for their ability to stabilize and deliver active compounds in both biomedical and food systems [4]. Recent advancements in encapsulation techniques have focused on enhancing the functionality of biopolymer matrices. Ultrasound-assisted modification has emerged as an effective tool to improve polymer performance by inducing chain depolymerization and promoting homogeneity in gel formation [5]. Compared to other encapsulation strategies such as nanoemulsions, spray drying, and electrospinning, ultrasound-assisted ionotropic gelation offers several advantages: it is energy-efficient, operates under mild conditions suitable for thermolabile compounds, and yields uniform microcapsules without requiring high-temperature drying or chemical crosslinkers [6]. While spray drying is cost-effective for large-scale production, it may compromise essential oil integrity due to thermal degradation. Nanoemulsion-based systems provide excellent bioavailability but often require surfactants and stabilizers, which may limit food-grade applications [7]. In contrast, ionotropic gelation with pectin is simple, food-safe, and compatible with clean label requirements, making it particularly suitable for functional food applications [8,9,10]. At the same time, growing interest in active food packaging, particularly for minimally processed (IV gamma) products, has led to the development of systems capable of extending shelf life by incorporating antimicrobial and antioxidant agents. Innovative materials such as nanocomposites [11], biomimetic films [12], and self-healing coatings [13] are being explored for these purposes. Nonetheless, pectin-based encapsulation remains a cost-effective and biodegradable solution that fits with circular economy principles and international sustainability goals, such as those outlined in the UN Sustainable Development Goals (SDG 12: Responsible Consumption and Production) [14,15]. This study aims to: (i) extract and characterize the chemical profile of LEO from Citrus limon (Eureka variety), (ii) evaluate its antioxidant and antifungal properties, (iii) optimize encapsulation using pectin via ionotropic gelation with and without ultrasound pretreatment, and (iv) test the application of the encapsulated oil on fresh-cut apples as a food model to assess its efficacy in mitigating browning, microbial growth, and quality deterioration during cold storage (Figure 1). The novelty of the study lies in its integrated approach combining bioactivity profiling, ultrasound-enhanced encapsulation, and real food application assessment under refrigerated conditions.

## 2. Materials and Methods

### 2.1. Plant Material

Lemon fruits (*Citrus limon*, Eureka variety) were harvested at maturity in March 2024 from the Constantine region of Algeria. Fruits were washed with sterile distilled water and manually peeled. The zest (flavedo) was separated from the albedo using a sterile stainless-steel knife. The flavedo was used fresh for essential oil extraction. The moisture content of peels was determined by drying fresh peels (three replicates) in an oven at 70 °C until the weight remained unchanged at 78.1 g/100 g of the total weight. The albedo was dried in a ventilated oven at 60 °C for 48 h, then finely ground (particle size < 500 µm) using a Moulinex^®^ (Ecully, France) electric grinder. Ground albedo was stored in airtight containers at room temperature until pectin extraction.

### 2.2. Essential Oil Extraction via Hydrodistillation

One hundred grams of fresh zest were subjected to hydrodistillation using a Clevenger-type apparatus for 3 h, with a peel-to-water ratio of 1:3 (*w*/*v*). The condenser temperature was maintained at 10 °C using a refrigerated water bath. The recovered essential oil was collected, dried over anhydrous sodium sulfate, and stored in amber glass vials at 4 °C. EO yield was calculated using Equation (1) [16]:

EO Yield (%) = (Weight of extracted oil/Weight of lemon zest) × 100(1)

### 2.3. Gas Chromatography–Mass Spectrometry (GC-MS) Analysis

The chemical composition of the essential oil was determined using an Agilent 6890N GC system equipped with a flame ionization detector (FID) and an HP-5 capillary column (30 m × 0.32 mm, 0.25 µm film). The injector temperature was set at 250 °C, with helium as the carrier gas at a constant flow of 1.0 mL/min. Oven temperature was programmed from 45 °C (hold 8 min) to 250 °C at 8 °C/min, then held for 10 min. Injection volume was 0.1 µL (split mode 1:20). Mass spectra were recorded at 70 eV and 280 °C interface temperature. Retention indices were calculated using n-alkane standards (C8–C30), and compounds were identified by comparison with NIST/Wiley libraries and literature data [17,18].

### 2.4. Antibacterial Activity Assay

Antibacterial activity was assessed by the disk diffusion method. Mueller–Hinton agar plates were inoculated with 18 h cultures of *Escherichia coli*, *Salmonella* spp., *Bacillus cereus*, and *Staphylococcus aureus*. Sterile filter paper disks (4 mm) were impregnated with 5 μL of EO and placed on the inoculated agar. Plates were incubated at 37 °C for 24 h. Gentamicin (10 µg/disc) served as the positive control, while DMSO (5 μL/disc) was the negative control. Inhibition zones were measured in mm (*n* = 3) [19].

### 2.5. Antifungal Activity Assay

The antifungal activity of LEO was evaluated against *Fusarium oxysporum*, *Aspergillus niger*, *Botrytis* spp., and *Penicillium* spp. using a poisoned food technique. Potato Dextrose Agar (PDA) was supplemented with EO at three concentrations: 0.075%, 0.15%, and 0.3% (*v*/*v*), prepared by dissolving the oil in 0.5% (*v*/*v*) DMSO. Sterile Petri dishes were poured with 20 mL of the PDA-EO mixture. Control groups included PDA with 0.5% DMSO (positive control) and PDA without any additives (negative control). A 5 mm diameter mycelial plug from a 6-day-old fungal culture was placed in the center of each plate. Plates were incubated at 25 °C for 6 days. The diameter of fungal growth was measured, and inhibition was calculated as (Equation (2)):

% Inhibition = [(C − T)/C] × 100(2)

where C is the colony diameter in the control and T is the diameter in the treated sample (*n* = 3) [19].

### 2.6. DPPH Radical Scavenging Assay

The DPPH assay was conducted following the method of Kerbab et al. [20], with modifications. A 0.1 mM solution of DPPH in ethanol was prepared and kept in the dark. For the test, 160 μL of DPPH solution was mixed with 40 μL of EO solution at varying concentrations (12.5–200 µg/mL). After 30 min of incubation in the dark at room temperature, absorbance was read at 517 nm using a UV-Vis spectrophotometer. Radical scavenging activity was calculated using Equation (3):

% Scavenging = [(C − T)/C] × 100(3)

where C is the absorbance of the control and T is the absorbance of the sample (*n* = 3).

### 2.7. ABTS Radical Scavenging Assay

The ABTS radical cation (ABTS^•+^) was generated by mixing 7 mM ABTS with 2.45 mM potassium persulfate and incubating in the dark at room temperature for 12–16 h. The working solution was diluted with ethanol to obtain an absorbance of 0.700 ± 0.020 at 734 nm. A volume of 160 µL ABTS solution was mixed with 40 µL of EO sample and incubated for 10 min. Absorbance was measured at 734 nm. The assay was carried out in triplicate [20].

### 2.8. Ferric Reducing Antioxidant Power (FRAP) Assay

The FRAP assay was performed according to Laghezza Masci et al. [21]. Briefly, 10 μL of EO solution was mixed with 40 μL of 0.2 M phosphate buffer (pH 6.6) and 50 μL of 1% potassium ferricyanide. The mixture was incubated at 50 °C for 20 min. Then, 50 μL of 10% trichloroacetic acid was added, followed by 40 μL of distilled water and 10 μL of 0.1% ferric chloride. Absorbance was recorded at 700 nm.

### 2.9. Phenanthroline Assay

Following the method of Szydłowska-Czerniak et al. [22], 10 μL of EO was mixed with 50 μL FeCl_3_ (0.2%), 30 μL phenanthroline (0.5%), and 110 μL methanol. After 20 min of incubation in the dark at 30 °C, absorbance was measured at 510 nm. BHT was used as a reference antioxidant.

### 2.10. Silver Nanoparticle (SNP) Assay

Silver nanoparticles were synthesized by reducing 1 mM AgNO_3_ with 1% trisodium citrate. A pale-yellow solution indicated the formation of SNPs. To assess antioxidant activity, 20 μL of EO was mixed with 130 μL SNP solution and 50 μL distilled water. After 30 min, absorbance was read at 423 nm [23].

### 2.11. Encapsulation Process via Ionotropic Gelation

A 2% (*w*/*v*) pectin solution was prepared in distilled water and stirred at 600 rpm for 30 min at room temperature. To investigate the impact of sonication, half of the solution was subjected to ultrasound treatment using a VWR ultrasonic bath (40 kHz, 100 W) for 10 min, while the other half remained untreated. Lemon essential oil (LEO) was incorporated into both solutions at 10% (*v*/*v*), and emulsification was achieved using a high-speed homogenizer (12,000 rpm for 3 min) to promote uniform droplet dispersion. The emulsions were transferred dropwise via a 5 mL syringe fitted with a 0.5 mm stainless-steel needle into a gently stirred 2% (*w*/*v*) calcium chloride (CaCl_2_) solution [24]. Ionotropic gelation occurred upon contact, forming spherical microcapsules that were allowed to harden for 30 min. Capsules were then filtered, rinsed with deionized water to remove excess ions, and air-dried at ambient temperature (22–25 °C) for 24 h. Encapsulation efficiency (EE) was evaluated by dissolving 10 mg of microcapsules in 4 mL of 2 M HCl, followed by thermal hydrolysis at 95 °C for 30 min. After cooling to room temperature, 2 mL of ethanol was added, and the solution was centrifuged at 9000 rpm for 5 min. The absorbance of the supernatant was measured at 275 nm using a UV-visible spectrophotometer, and EO content was quantified against a calibration curve of known LEO concentrations. Encapsulation efficiency was calculated using Equation (4):

EE (%) = (Encapsulated oil/Initial oil) × 100(4)

### 2.12. Scanning Electron Microscopy (SEM)

The morphology and surface features of dried microcapsules were analyzed using a Quanta 250 Scanning Electron Microscope (FEI Company, Hillsboro, OR, USA) equipped with a tungsten filament. Capsules were fixed on aluminum stubs using double-sided conductive carbon tape. No gold sputter coating was applied in order to observe the native surface morphology, following the method by Almeida et al. [25].

The SEM was operated in low-vacuum mode at an accelerating voltage of 10–15 kV. Images were acquired at magnifications ranging from 250× to 2500×. Microcapsule diameter was measured on 100 individual particles using ImageJ software ((version 1.54f)). Capsule morphology was analyzed using a Quanta 250 SEM (FEI) equipped with a tungsten filament, operated at an accelerating voltage of 20 kV.

### 2.13. Preparation of Apple Slices and Treatments

Fresh-cut apple slices were selected as the food model for testing the preservative efficacy of the encapsulated lemon essential oil (LEO). This model was chosen due to its high susceptibility to enzymatic browning and microbial spoilage, making it suitable for evaluating antioxidant and antimicrobial performance in a real food matrix. Uniform Golden Delicious apples (*Malus domestica* Borkh), weighing 160–180 g, were selected based on size, absence of bruises, and ripeness (firmness > 4.0 N). Fruits were washed under tap water, sanitized with 0.5% sodium hypochlorite for 2 min, rinsed with sterile distilled water, and air-dried. Apples were peeled with a sterile stainless-steel knife and sliced into uniform rectangular pieces (4 × 1 × 1 cm).

Slices were randomly divided into three groups:

- Control (C1): untreated;
- Blank capsules (C2): immersed for 1 min in a 2% (*w*/*v*) suspension of empty pectin capsules;
- LEO capsules (C3): immersed for 1 min in a 2% (*w*/*v*) suspension of EO-loaded capsules.

Capsules (2 g) were dispersed in 100 mL sterile distilled water and stirred at 300 rpm for 10 min to ensure uniform distribution. After immersion, slices were left to dry in a laminar flow cabinet (ISO Class 5) for 5 min. Samples were then packed (3 slices per container) in sterile, food-grade polyethylene boxes (250 mL) and stored at 4 ± °C and 75% relative humidity. Analyses were conducted on days 0, 3, 6, and 10.

### 2.14. Color and Visual Browning Assessment

Instrumental color measurement was performed using a CR-5 Konica Minolta colorimeter (Minolta Co., Osaka, Japan) calibrated with a white standard tile. For each slice, three readings were taken from different positions on the cut surface, and mean values for L* (lightness), a* (red-green axis, indicating redness), and b* (blue-yellow axis, indicating yellowness) were recorded [26].

Visual browning was independently scored by two trained evaluators using a five-point scale:

0 = no browning;

1 = slight (<25% of surface);

2 = moderate (25–50%);

3 = extensive (50–75%);

4 = severe (>75%).

Discrepancies between assessors greater than 1 point were resolved by joint re-evaluation. Images were captured with a high-resolution DSLR camera(Nikon D3500 (Nikon Corporation, Tokyo, Japan) at each time point under standardized lighting conditions.

### 2.15. Microbial Load Evaluation

Microbial analyses were performed at each storage point (0, 3, 6, and 10 days). Three apple slices per treatment were transferred aseptically into sterile stomacher bags containing 90 mL of buffered peptone water (BPW, Oxoid) and homogenized for 2 min using a BagMixer^®^ 400P lab blender (Interscience, Saint Nom la Breteche, France).

Serial decimal dilutions were plated in duplicate on the following:

- Plate Count Agar (PCA) for total mesophilic aerobic bacteria (37 °C, 48 h);
- Potato Dextrose Agar (PDA) with 1% tartaric acid for yeasts and molds (25 °C, 72 h).

Results were expressed as mean log CFU per gram of sample. Plates with 25–250 colonies were selected for enumeration.

### 2.16. Weight Loss and Firmness

Each apple slice was weighed at time 0 (W_0_) and on days 3, 6, and 10 (Wt) using a precision balance (±0.001 g; OHAUS Pioneer PX, Parsippany, NJ, USA). Weight loss (%) was calculated using Equation (5):

Weight loss (%) = [(W_0_ − Wt)/W_0_] × 100 (5)

where W_0_ is the initial weight and W_t_ is the weight at time t.

Firmness was evaluated using a digital texture analyzer (Model TR Snc., Forlì, Italy) equipped with an 8 mm cylindrical probe. Penetration was performed to a depth of 5 mm at a speed of 1 mm/s. Measurements were taken at two opposing sides of the slice and averaged (*n* = 3 per treatment).

### 2.17. Statistical Analysis

All measurements were performed in triplicate and expressed as mean ± standard deviation (SD). Normality was checked using the Shapiro–Wilk test. One-way ANOVA followed by Tukey’s HSD post hoc test was used to determine significant differences among treatments (*p* < 0.05). For non-parametric data, the Kruskal–Wallis test was applied. Microbial data were log-transformed before analysis. All statistical tests were conducted using IBM SPSS Statistics v26.0 (IBM Corp., Armonk, NY, USA).

## 3. Results

### 3.1. Chemical Composition of Lemon Essential Oil

Hydrodistillation of Citrus limon (Eureka variety) peel yielded 3.85% essential oil (LEO). GC-MS analysis identified 31 compounds, accounting for over 95% of the total oil composition. The major constituents were limonene (56.18%), β-pinene (9.89%), γ-terpinene (9.75%), β-bisabolene (3.01%), α-pinene (2.89%), and β-myrcene (2.54%) (Table 1). This composition is in agreement with previous studies on lemon EO from Mediterranean cultivars, confirming a typical monoterpene-rich chemotype [27]. Limonene, the predominant component, is widely recognized for its antioxidant and antimicrobial activities. Similar concentrations have been reported in Italian and Turkish lemon varieties, reinforcing the consistency of chemotypes across growing regions [27,28]. The presence of γ-terpinene and β-pinene further supports the expected bioactivity profile, as these monoterpenes contribute synergistically to both radical-scavenging and antimicrobial effects [29]. The high limonene content suggests strong membrane-disruptive potential, particularly against Gram-positive bacteria. Literature reports indicate that limonene-rich EOs destabilize bacterial membranes, causing leakage of intracellular ions and ATP [30]. This mechanism is consistent with the antimicrobial results presented in Section 3.2.

### 3.2. Antibacterial Activity

**Figure 2:**
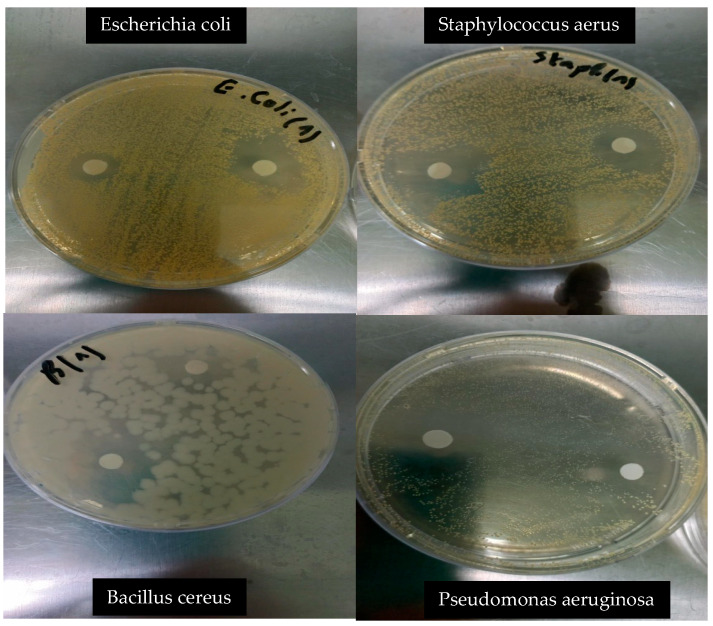
Antibacterial activity of lemon essential oil against selected bacterial strains.

The lemon essential oil (LEO) exhibited notable antibacterial activity against both Gram-positive and Gram-negative bacteria (Table 2). Among the tested strains, *Bacillus cereus* and *Staphylococcus aureus* showed the highest sensitivity, with inhibition zones of 29.1 ± 1.15 mm and 26.6 ± 1.60 mm, respectively. In contrast, *Escherichia coli* (16.5 ± 0.41 mm) and *Salmonella* spp. (9.0 ± 0.86 mm) were moderately inhibited, indicating a differential response based on bacterial cell wall structure (Figure 2). These results are consistent with previous studies that reported higher susceptibility of Gram-positive bacteria to monoterpene-rich essential oils [31]. The high inhibition zones observed for *B. cereus* and *S. aureus* can be attributed to the hydrophobic nature of limonene, γ-terpinene, and β-pinene, which enables diffusion through the thick peptidoglycan layer of Gram-positive cell walls, leading to membrane destabilization, ion leakage, and ATP depletion [30]. While Gram-negative bacteria are generally more resistant due to the presence of an outer membrane, the moderate activity observed against *E. coli* suggests that LEO still possesses a degree of broad-spectrum efficacy. These findings support the potential application of lemon EO in antimicrobial formulations, particularly for targeting Gram-positive spoilage or pathogenic bacteria in food systems.

### 3.3. Antifungal Activity

Lemon essential oil (LEO) exhibited dose-dependent antifungal activity against four phytopathogenic fungi: *Fusarium oxysporum*, *Aspergillus niger*, *Botrytis* spp., and *Penicillium* spp. (Table 3). At a concentration of 0.3%, the highest inhibition was observed against *Botrytis* spp. (59.3%) and *A. niger* (58.18%), followed by *Penicillium* spp. (40.0%) and *F. oxysporum* (15.38%). Decreasing the EO concentration resulted in a progressive reduction in antifungal efficacy, with the weakest activity recorded at 0.075%. These findings are in agreement with prior studies reporting greater susceptibility of *Botrytis* and *Aspergillus* species to monoterpene-rich essential oils [32,33,34]. The differential sensitivity among fungal strains may be attributed to variations in cell wall permeability, ergosterol content, and oxidative stress defenses. Compounds such as limonene and γ-terpinene are known to disrupt fungal membranes and induce intracellular reactive oxygen species (ROS) accumulation, leading to cellular dysfunction and growth inhibition [35]. Furthermore, the observed antifungal activity may be potentiated by synergistic effects between limonene and other minor components, including myrcene and bisabolene [36]. Such interactions have been suggested to enhance the fungistatic or fungicidal capacity of citrus-derived essential oils, particularly under controlled storage conditions. These results support the potential use of LEO as a natural antifungal agent in post-harvest preservation or biofilm disruption applications.

### 3.4. Antioxidant Activity

Lemon essential oil (LEO) demonstrated moderate to strong antioxidant activity across five complementary assays (Table 4). The IC_50_ values for radical scavenging were 28.43 ± 0.14 µg/mL in the DPPH assay and 35.01 ± 0.11 µg/mL in the ABTS assay, indicating a capacity to neutralize free radicals via hydrogen atom transfer mechanisms. The reducing power, measured via the FRAP assay (A_0 5_= 66.59 ± 0.45 µg/mL), and the phenanthroline method (A_0 5_ = 187.84 ± 0.34 µg/mL), reflected the presence of electron-donating compounds, though to a lesser extent than observed in synthetic antioxidants. Interestingly, the SNP assay revealed a metal-chelating capacity (A_0 5_ = 29.67 ± 0.05 µg/mL), suggesting that LEO components may contribute to lipid peroxidation inhibition by binding transition metals. Compared to standard antioxidants such as BHT, BHA, and Trolox, LEO exhibited lower potency, particularly in electron transfer-based systems. However, the performance was consistent with previous reports on citrus essential oils, where antioxidant activity is primarily associated with monoterpenes like limonene and γ-terpinene [29]. These compounds, although less effective than polyphenols in single-electron transfer reactions, contribute meaningfully through hydrogen donation and metal ion chelation. Notably, the SNP assay was applied for the first time to lemon EO in this study and highlighted a distinct antioxidant mechanism compared to DPPH or ABTS. The relatively weaker effects observed in FRAP and phenanthroline assays may reflect the chemical nature of EO constituents, which lack the polyphenolic structures typical of strong reducing agents [37,38]. Overall, the antioxidant profile of LEO reflects a multifaceted mechanism, with varying contributions from radical scavenging, metal binding, and moderate reducing power, supporting its potential use in oxidative stress mitigation in food or cosmetic applications.

### 3.5. Encapsulation Efficiency

**Figure 3:**
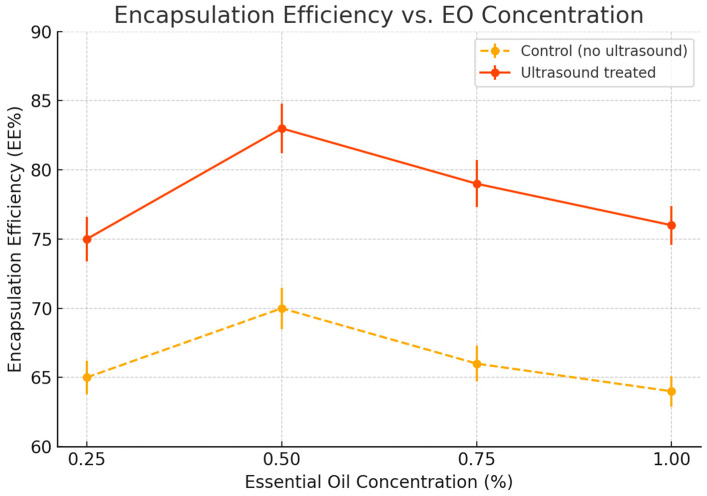
Encapsulation efficiency (EE%) of lemon essential oil in pectin-based microcapsules as a function of essential oil concentration (0.25%, 0.50%, 0.75%, 1.00%), with and without ultrasonic pretreatment. Values are expressed as mean ± standard deviation (*n* = 3). Error bars indicate standard deviation.

**Figure 4:**
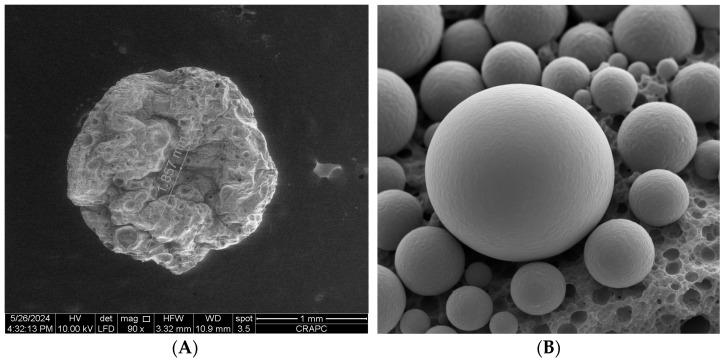
Scanning Electron Micrographs (SEMs) of pectin-based microcapsules containing lemon essential oil. (**A**) Microcapsules obtained without ultrasound pretreatment: irregular shape with porous and wrinkled surface. (**B**) Microcapsules obtained with ultrasound pretreatment: smooth, spherical, and compact morphology with uniform surface, indicating improved gelation and encapsulation efficiency. Scale bars are included for dimensional reference.

**Figure 5:**
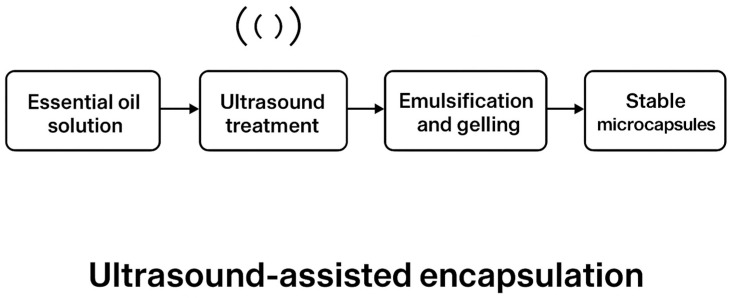
Schematic representation of ultrasound-assisted encapsulation via ionotropic gelation. Ultrasound promotes pectin chain depolymerization, enhances oil droplet emulsification, and facilitates homogeneous gelation in calcium solution, resulting in compact, spherical microcapsules with improved encapsulation efficiency.

Encapsulation efficiency (EE) was significantly affected by ultrasound pretreatment. The highest EE value (82.3%) was obtained at 0.5% essential oil concentration in the sonicated samples, compared to 68.5% in the non-treated control (Figure 3). This suggests that ultrasound enhances entrapment capacity, likely by increasing the mobility of pectin chains and improving emulsification of essential oil droplets. Ultrasonic cavitation promotes partial depolymerization of pectin and improves solubility, facilitating tighter crosslinking with calcium ions during ionotropic gelation [39,40]. This process reduces surface oil loss and phase separation, resulting in more efficient and stable encapsulation. The mechanism underlying ultrasound-assisted encapsulation is attributed to acoustic cavitation, which generates localized shear forces and microstreaming effects [41]. These mechanical actions disrupt polymeric aggregates, improving matrix homogeneity and droplet distribution [42]. Ultrasound also enhances the emulsification of the oil phase, producing smaller and more uniform droplets that are more effectively entrapped. Similar findings have been reported for ultrasound-treated alginate and chitosan systems, where improved EE%, surface morphology, and structural uniformity were observed [43]. Scanning electron microscopy (Figure 4) confirmed morphological differences between the two capsule types. Control microcapsules were irregular, wrinkled, and porous, whereas those treated with ultrasound were smooth, spherical, and compact, reflecting improved network cohesion and gelation uniformity. Particle sizes ranged from 110 to 400 µm, with more consistent diameters in the ultrasound group (280 ± 40 µm) versus the control (310 ± 60 µm). This size uniformity likely results from improved oil droplet dispersion and consistent gelling kinetics during encapsulation. Notably, the absence of surface pores in either group suggests that oil release occurs via slow diffusion or matrix erosion rather than sudden rupture. This supports the potential of ultrasound-treated capsules for controlled release applications, reducing the risk of burst release and unwanted oil migration when applied to food surfaces such as fresh-cut apples. The overall mechanism of ultrasound-assisted encapsulation is summarized in Figure 5, which highlights the role of cavitation in promoting chain flexibility, droplet dispersion, and efficient gelation. Future work will investigate release behavior and stability during long-term refrigerated storage.

### 3.6. Evaluation of EO-Loaded Pectin Microcapsules in a Food Model

**Figure 6:**
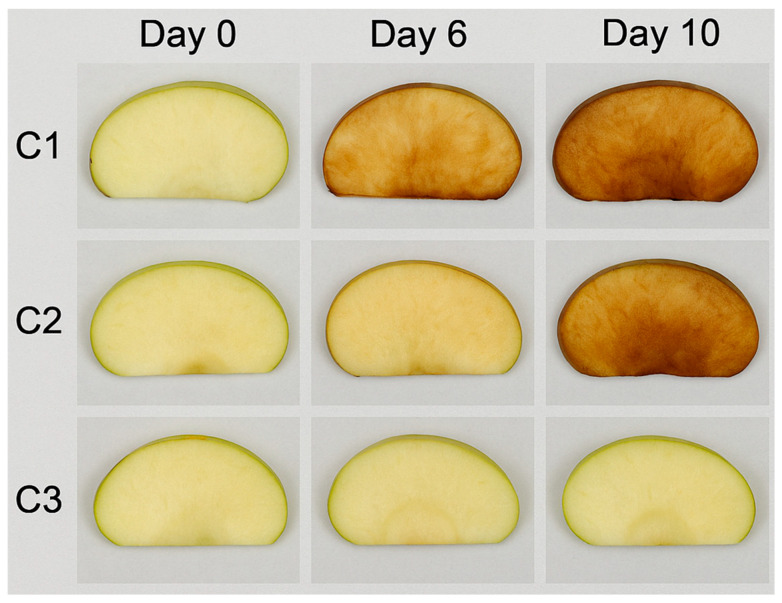
Visual evaluation of fresh-cut apple slices stored at 4 °C for 10 days under different treatments. C1: untreated control; C2: coated with empty pectin capsules; C3: coated with pectin capsules containing lemon essential oil. Time points include Day 0, Day 6, and Day 10.

The application of LEO-loaded pectin microcapsules (C3) to fresh-cut apples significantly improved quality preservation during refrigerated storage. On Day 6, treated slices showed markedly reduced browning, with a visual score of 1.2 ± 0.3, compared to 2.8 ± 0.2 in the untreated control (C1). By Day 10, L* values remained higher in C3 (69.3 ± 1.1) than in both control (59.4 ± 1.8) and pectin-only (C2) groups, reflecting better visual appearance and reduced oxidation (Table 5; Figure 3). These effects are attributed to the antioxidant capacity of LEO, particularly its ability to suppress polyphenol oxidase (PPO) activity and inhibit reactive oxygen species (ROS) formation, consistent with previous findings [44,45]. The a* values (redness) increased in all samples during storage but were significantly lower in C3 (3.2 ± 0.3), confirming slower browning kinetics. Although b* values were recorded, they showed no statistical significance and were therefore omitted from detailed discussion. The EO’s antimicrobial properties also contributed to lower microbial loads in C3-treated slices, with aerobic bacteria reduced to 4.9 ± 0.2 log CFU/g and fungal counts to 3.1 ± 0.3 log CFU/g, consistent with earlier studies that demonstrated microbial inhibition through citrus EO-infused films [19,46]. Moreover, weight loss was lowest in the C3 group (4.2% vs. 6.4% in controls), likely due to the moisture barrier effect of the encapsulated pectin matrix. This structural protection also contributed to superior firmness retention in C3 (2.3 ± 0.1 N), suggesting reduced enzymatic degradation during storage. These outcomes reinforce the dual functionality of the LEO-loaded capsules, as both a physical barrier and a bioactive delivery system. This is supported by the improved preservation parameters across all measured indices and agrees with the literature on EO-based coatings for IV gamma and post-harvest products [47,48]. While the respiration rate was not measured in this study, the lower browning, moisture loss, and microbial activity suggest a slowed metabolic decay, validating the effectiveness of this encapsulation strategy in real food matrices. The visual preservation observed in Figure 6, particularly the reduced browning in the C3 group, reflects the effective suppression of enzymatic oxidation, likely due to the inhibition of polyphenol oxidase (PPO) and mitigation of reactive oxygen species (ROS) by bioactive compounds in lemon essential oil. Similar visual protection effects using citrus EO in minimally processed fruit systems have been reported [49]. Although the respiration rate was not directly measured in this study, the reduced browning and lower microbial load suggest a slowed metabolic deterioration, which has been previously associated with reduced oxygen consumption and ethylene production in EO-treated fresh-cut produce [50]. Future investigations will aim to quantify respiration parameters and PPO activity to better correlate visual preservation with underlying physiological processes.

## 4. Conclusions

This study confirmed the potential of lemon essential oil (LEO) from Citrus limon (Eureka variety) as a natural antioxidant and antifungal agent and demonstrated its effective stabilization through ultrasound-assisted pectin encapsulation. The optimized microcapsules exhibited high encapsulation efficiency, uniform morphology, and enhanced preservation effects in a fresh-cut apple model, significantly reducing browning, microbial growth, and moisture loss during storage. These results support the use of ultrasound-assisted pectin gelation as a scalable strategy for developing bioactive delivery systems applicable in food preservation. Further studies are warranted to evaluate release behavior, regulatory safety, and applicability in diverse food matrices.

## Figures and Tables

**Table 1:** Chemical composition of lemon (Citrus limon, Eureka variety) essential oil as determined by GC-MS analysis.

No.	RT (Min)	Compound Name	LRI	Area (%) ± SD
1	7.984	1-Phellandrene	714	0.67 ± 0.02
2	8.302	α-Pinene	930	2.82 ± 0.08
3	9.156	Camphene	968	0.12 ± 0.00
4	11.198	β-Pinene	982	9.89 ± 0.30
5	12.122	Bicycloheptane dimethyl methylene	1015	0.03 ± 0.00
6	12.640	β-Myrcene	1020	2.54 ± 0.08
7	15.978	L-Limonene	1030	56.18 ± 1.69
8	17.002	D-Limonene	1032	1.56 ± 0.05
9	17.596	γ-Terpinene	1064	9.75 ± 0.29
10	19.450	α-Terpinolene	1085	0.58 ± 0.02
11	21.234	Cyclohexene methyl	1132	0.02 ± 0.00
12	22.723	cis-Limonene oxide	1131	0.43 ± 0.01
13	23.106	Spiroheptane derivative	1180	0.47 ± 0.01
14	23.653	4,8-Epoxy-p-menth-1-ene	1200	0.14 ± 0.00
15	24.542	Citronellal	1235	0.17 ± 0.01
16	26.449	3-Cyclohexen-1-ol derivative	1250	0.02 ± 0.00
17	28.374	Cyclohexene methanol	1300	0.12 ± 0.00
18	30.775	Z-Citral	1320	1.60 ± 0.05
19	32.971	Z-Citral/Cis-Citral	1350	2.19 ± 0.07
20	38.333	Tetramethylcyclopentenone	1390	0.62 ± 0.02
21	39.069	cis-Neryl acetate	1410	1.94 ± 0.06
22	40.346	Neryl/Geranyl acetate	1430	1.49 ± 0.04
23	41.659	Isocaryophyllene	1450	0.93 ± 0.03
24	42.989	trans-α-Bergamotene	1470	1.77 ± 0.05
25	44.631	trans-β-Farnesene	1400	0.20 ± 0.01
26	45.962	Cyclohexene derivative	1510	0.06 ± 0.00
27	46.356	Valencene	1530	0.14 ± 0.00
28	47.351	cis-α-Bisabolene	1540	0.12 ± 0.00
29	47.680	β-Bisabolene	1570	3.01 ± 0.09
30	49.670	trans-α-Bisabolene	1590	0.06 ± 0.00
31	51.518	Octahydro-inden-4-ol	1600	0.33 ± 0.01

Area values are expressed as mean percentage ± standard deviation (SD), (*n* = 3).

**Table 2:** Antibacterial activity of lemon essential oil against selected bacterial strains, measured by the agar diffusion method.

Microorganism	Inhibition Zone (mm)	Sensitivity Level
*Escherichia coli*	16.5 ± 0.41	++
*Salmonella* spp.	9.0 ± 0.86	+
*Bacillus cereus*	29.1 ± 1.15	+++
*Staphylococcus aureus*	26.6 ± 1.60	+++

Results are expressed as mean inhibition zone diameter ± standard deviation (mm); *n* = 3.

**Table 3:** Antifungal activity of lemon essential oil at different concentrations against common phytopathogenic fungi.

EO Concentration	*F. oxysporum*	*A. niger*	*Botrytis* spp.	*Penicillium* spp.
0.3%	15.38% ± 0.9%	58.18% ± 2.4%	59.3% ± 1.9%	40.0% ± 2.0%
0.15%	10.66% ± 0.7%	34.54% ± 1.4%	44.18% ± 1.5%	31.42% ± 1.2%
0.075%	5.33% ± 0.4%	31.54% ± 1.3%	39.53% ± 1.2%	20.0% ± 0.8%

Values are expressed as mean ± standard deviation (*n* = 3). Growth inhibition was measured after 6 days of incubation.

**Table 4:** Antioxidant activity of lemon essential oil evaluated by five different assays.

Sample	DPPH IC_50_	ABTS IC_50_	Phenanthroline A_0 5_	FRAP A_0 5_	SNP A_0 5_
Lemon EO	28.43 ± 0.14	35.01 ± 0.11	187.84 ± 0.34	66.59 ± 0.45	29.67 ± 0.05
BHA	5.99 ± 0.64	1.83 ± 0.67	0.94 ± 0.05	ND	ND
BHT	12.99 ± 0.41	1.53 ± 0.40	2.29 ± 0.10	ND	ND
α-Tocopherol	ND	ND	ND	35.26 ± 0.39	ND
Trolox	ND	ND	ND	ND	34.17 ± 1.23

Results are expressed as IC_50_ or A_0 5_ (µg/mL). Lower values indicate higher antioxidant activity. ND: Not detected.

**Table 5:** Effect of pectin-based lemon EO microcapsules on the quality parameters of fresh-cut apples during refrigerated storage at 4 °C.

Parameter	C1 (Control)	C2 (Pectin Only)	C3 (Pectin + EO)
Browning Score (Day 6)	2.8 ± 0.2 ^a^	2.3 ± 0.2 ^b^	1.2 ± 0.3 ^c^
L* (Lightness, Day 10)	59.4 ± 1.8 ^a^	63.8 ± 1.6 ^b^	69.3 ± 1.1 ^c^
a* (Redness, Day 10)	5.6 ± 0.4 ^a^	4.4 ± 0.3 ^b^	3.2 ± 0.3 ^c^
b* (Yellowness, Day 10)	22.6 ± 0.6 ^a^	23.0 ± 0.5 ^a^	23.4 ± 0.4 ^a^
Aerobic Bacteria (log CFU/g, Day 10)	6.5 ± 0.4 ^a^	5.8 ± 0.3 ^b^	4.9 ± 0.2 ^c^
Fungal Count (log CFU/g, Day 10)	4.4 ± 0.3 ^a^	3.6 ± 0.2 ^b^	3.1 ± 0.3 ^c^
Weight Loss (%, Day 10)	6.4 ± 0.5 ^a^	5.1 ± 0.4 ^b^	4.2 ± 0.3 ^c^
Firmness (N, Day 10)	1.4 ± 0.2 ^a^	1.9 ± 0.2 ^b^	2.3 ± 0.1 ^c^

Values are presented as mean ± SD (*n* = 3). Different superscript letters within the same row indicate significant differences (*p* < 0.05) based on Tukey’s test. Values marked with an asterisk (L*, a*, b*) refer to CIE color parameters: L* = lightness, a* = redness, and b* = yellowness.

## Data Availability

The original contributions presented in the study are included in the article; further inquiries can be directed to the corresponding author.

## References

[1] Koch P., Dhua S., Mishra P. (2024). Critical review on Citrus essential oil extracted from processing waste-based nanoemulsion: Preparation, characterization, and emerging food application. J. Essent. Oil Res..

[2] Fernandes B., Oliveira M.C., Marques A.C., Dos Santos R.G., Serrano C. (2024). Microencapsulation of Essential Oils and Oleoresins: Applications in Food Products. Foods.

[3] Said N.S., Olawuyi I.F., Lee W.Y. (2023). Pectin hydrogels: Gel-forming behaviors, mechanisms, and food applications. Gels.

[4] Freitas C.M.P., Coimbra J.S.R., Souza V.G.L., Sousa R.C.S. (2021). Structure and applications of pectin in food, biomedical, and pharmaceutical industry: A review. Coatings.

[5] Qayum A., Rashid A., Liang Q., Wu Y., Cheng Y., Kang L., Liu Y., Zhou C., Hussain M., Ashokkumar M. (2023). Ultrasonic and homogenization: An overview of the preparation of an edible protein–polysaccharide complex emulsion. Compr. Rev. Food Sci. Food Saf..

[6] Kaur G., Khan Z.S., Toker Ö.S., Bhat M.S., Basyigit B., Kurt A., Rustagi S., Suri S., Hatami S., Fayaz S. (2024). Innovative approaches to pectin processing: Enhancing techno-functional properties for applications in food and beyond. Bioact. Carbohydr. Diet. Fibre.

[7] Das P., Panda J.R., Patro C.N., Sahu B., Patnaik S.S. (2023). A comprehensive review of Nanoemulsion applications and their recent advancements. Curr. Nanomater..

[8] Feng L., Wang Y., Liu T., Zhao C., Chen Y., Wang F., Bao Y., Zheng J. (2024). Pectin-based emulsion gels prepared by acidic and ionotropic methods for intestinal targeted delivery in vitro. Food Hydrocoll..

[9] Silva N.C., Chevigny C., Domenek S., Almeida G., Assis O.B.G., Martelli-Tosi M. (2025). Nanoencapsulation of active compounds in chitosan by ionic gelation: Physicochemical, active properties and application in packaging. Food Chem..

[10] Ishwarya S.P., Nisha P. (2022). Advances and prospects in the food applications of pectin hydrogels. Crit. Rev. Food Sci. Nutr..

[11] Kehinde B.A., Chhikara N., Sharma P., Garg M.K., Panghal A. (2021). Application of polymer nanocomposites in food and bioprocessing industries. Handbook of Polymer Nanocomposites for Industrial Applications.

[12] Feng Z., Sun P., Zhao F., Li M., Ju J. (2024). Advancements and challenges in biomimetic materials for food preservation: A review. Food Chem..

[13] Lai W.F. (2023). Design and application of self-healable polymeric films and coatings for smart food packaging. npj Sci. Food.

[14] Kothapalli L., Nikam N., Thomas A., Bhikne N., Kamdi S. (2025). Pectin from Agro-waste to Utility Product. Curr. Green Chem..

[15] Panda J., Mishra A.K., Mohanta Y.K., Patowary K., Rauta P.R., Mishra B. (2024). Exploring biopolymer for food and pharmaceuticals application in the circular bioeconomy: An agro-food waste-to-wealth approach. Waste Biomass Valori..

[16] Tuan N.T., Dang L.N., Huong B.T.C., Danh L.T. (2019). One Step Extraction of Essential Oils and Pectin from Pomelo (Citrus grandis) Peels. Chem. Eng. Process. Process Intensif..

[17] Zellner B.D.A., Bicchi C., Dugo P., Rubiolo P., Dugo G., Mondello L. (2008). Linear retention indices in gas chromatographic analysis: A review. Flavour Frag. J..

[18] Zerrad H., Bakrim H., Moullamri M., Bakkali M., Alibrando F., Cacciola F., Mondello M., Dugo P., Mondello L., Arakrak A. (2024). Chemical screening, antimicrobial and antioxidant activities of Ruta angustifolia essential oil from Morocco. J. Essent. Oil Res..

[19] Akachat B., Himed L., Salah M., D’Elia M., Rastrelli L., Barkat M. (2025). Development of Pectin-Based Films with Encapsulated Lemon Essential Oil for Active Food Packaging: Improved Antioxidant Activity and Biodegradation. Foods.

[20] Kerbab K., Sanah I., Djeghim F., Belattar N., Santoro V., D’Elia M., Rastrelli L. (2025). Nutritional Composition, Physicochemical Properties, Antioxidant Activity, and Sensory Quality of Matricaria chamomilla-Enriched Wheat Bread. Foods.

[21] Laghezza Masci V., Mezzani I., Alicandri E., Tomassi W., Paolacci A.R., Covino S., Vinciguerra V., Catalani E., Cervia D., Ciaffi M. (2025). The Role of Extracts of Edible Parts and Production Wastes of Globe Artichoke (*Cynara cardunculus L*. var. scolymus (L.) in Counteracting Oxidative Stress. Antioxidants.

[22] Szydłowska-Czerniak A., Dianoczki C., Recseg K., Karlovits G., Szłyk E. (2008). Determination of Antioxidant Capacities of Vegetable Oils by Ferric-Ion Spectrophotometric Methods. Talanta.

[23] Özyürek M., Güngör N., Baki S., Güçlü K., Apak R. (2012). Development of a silver nanoparticle-based method for the antioxidant capacity measurement of polyphenols. Anal. Chem..

[24] Zhang D., Chen D., Campanella O.H. (2024). The Effect of CaCl_2_ on the Gelling Properties of Pea Protein–Pectin Dispersions. Gels.

[25] Almeida F.D.L., Genisheva Z., Oliveira J.M., Vicente A.A., Teixeira J.A. (2018). Pectin microparticles with lemon essential oil for antimicrobial applications. Food Bioprod. Process..

[26] Subhashree S.N., Sunoj S., Xue J., Bora G.C. (2017). Quantification of browning in apples using colour and textural features by image analysis. Food Qual. Saf..

[27] Modica G., Strano T., Napoli E., Seminara S., Aguilar-Hernández M., Legua P., Gentile A., Ruberto G., Continella A. (2024). Qualitative traits and peel essential oil profiles of 24 Italian and international lemon varieties. Food Biosci..

[28] Güzel B., Canlı O., Yüce B., Hocaoglu S.M. (2024). Determination of limonene chirality in oils obtained from different types of Citrus waste peels in Türkiye. J. Turk. Chem. Soc. A Chem..

[29] Guo Y., Baschieri A., Amorati R., Valgimigli L. (2021). Synergic antioxidant activity of γ-terpinene with phenols and polyphenols enabled by hydroperoxyl radicals. Food Chem..

[30] da Silva Martins L.H., da Silva S.B., Filho A.F., Komesu A., de Oliveira J.A.R., Moreira D.K. (2023). T Essential Oils Used to Inhibit Bacterial Growth in Food. Essential Oils: Extraction Methods and Applications.

[31] Aelenei P., Miron A., Trifan A., Bujor A., Gille E., Aprotosoaie A.C. (2016). Essential oils and their components as modulators of antibiotic activity against gram-negative bacteria. Medicines.

[32] Boukhatem M.N., Ferhat M.A., Kameli A., Saidi F., Mekarnia M.A. (2021). Lemon Essential Oil: Antifungal Activity and Mode of Action against Fusarium oxysporum f. sp. albedinis. J. Essent. Oil Res..

[33] Viuda-Martos M., Ruiz-Navajas Y., Fernández-López J., Pérez-Álvarez J. (2008). Antifungal activity of lemon (*Citrus lemon* L.), mandarin (*Citrus reticulata* L.), grapefruit (*Citrus paradisi* L.) and orange (*Citrus sinensis* L.) essential oils. Food Control.

[34] Singh P., Shukla R., Prakash B., Kumar A., Singh S., Dubey N.K. (2010). Chemical profile, antifungal, antiaflatoxigenic andantioxidant activity of Citrus limon essential oil and its efficacy as a preservative against fungal spoilage of dry fruits. LWT-Food Sci. Technol..

[35] Gao T., Zhou H., Zhou W., Hu L., Chen J., Shi Z. (2016). The fungicidal activity of thymol against Fusarium graminearum via inducing lipid peroxidation and disrupting ergosterol biosynthesis. Molecules.

[36] Rajčević N., Bukvički D., Dodoš T., Marin P.D. (2022). Interactions between natural products-A review. Metabolites.

[37] Ben Jemaa J.M., Tersim N., Khouja M.L., Ben Jilani L., Hamrouni I. (2012). Insecticidal and Antioxidant Activities of Essential Oils from *Citrus limon* (L.) Osbeck Leaves. Ind. Crops Prod..

[38] Bibi Sadeer N., Montesano D., Albrizio S., Zengin G., Mahomoodally M.F. (2020). The versatility of antioxidant assays in food science and safety, Chemistry, applications, strengths, and limitations. Antioxidants.

[39] Bachari S., Ghaderi-Ghahfarokhi M., Gavlighi H.A., Zarei M. (2024). Ultrasonic depolymerization of pomegranate peel pectin: Effect of sonication time on antioxidant, α-amylase inhibitory, and prebiotic properties. Food Chem. X.

[40] Larsen L.R., van der Weem J., Caspers-Weiffenbach R., Schieber A., Weber F. (2021). Effects of ultrasound on the enzymatic degradation of pectin. Ultrason. Sonochem..

[41] Vela A.J., Villanueva M., Ronda F. (2024). Ultrasonication: An efficient alternative for the physical modification of starches, flours and grains. Foods.

[42] Kumar A.R.S., Padmakumar A., Kalita U., Samanta S., Baral A., Singha N.K., Ashokkumar M., Qiao G.G. (2023). Ultrasonics in polymer science: Applications and challenges. Prog. Mater. Sci..

[43] Yang X., Lv Z., Han C., Zhang J., Duan Y., Guo Q. (2024). Stability and encapsulation properties of daidzein in zein/carrageenan/sodium alginate nanoparticles with ultrasound treatment. Int. J. Biol. Macromol..

[44] Dhanasekaran S., Liang L., Gurusamy S., Yang Q., Zhang H. (2024). Chitosan stabilized lemon essential oil nanoemulsion controls black mold rot and maintains quality of table grapes. Int. J. Biol. Macromol..

[45] Dhanasekaran S., Liang L., Gurusamy S., Godana E.A., Yang Q., Zhang H. (2025). Efficacy and mechanism of chitosan nanoparticles containing lemon essential oil against blue mold decay of apples. Int. J. Biol. Macromol..

[46] Li Y., Sun J., Ai C., Song S., Yang J. (2025). Edible Nano-Film Incorporated with Lemon Essential Oil-Loaded Pickering Emulsion for Cold Storage Improvement of Penaeus vannamei. Food Biosci..

[47] Sharma R., Nath P.C., Das P., Rustagi S., Sharma M., Sridhar N., Hazarika T.K., Rana P., Nayak P.K., Sridhar K. (2024). Essential oil-nanoemulsion based edible coating: Innovative sustainable preservation method for fresh/fresh-cut fruits and vegetables. Food Chem..

[48] Taban A., Haghighi T.M., Mousavi S.S., Sadeghi H. (2024). Are edible coatings (with or without essential oil/extract) game changers for maintaining the postharvest quality of strawberries? A meta-analysis. Postharvest Biol. Technol..

[49] Zdulski J.A., Rutkowski K.P., Konopacka D. (2024). Strategies to Extend the Shelf Life of Fresh and Minimally Processed Fruit and Vegetables with Edible Coatings and Modified Atmosphere Packaging. Appl. Sci..

[50] Akbari S., Radi M., Hosseinifarahi M., Amiri S. (2024). Microbial and physicochemical changes in green bell peppers treated with ultrasonic-assisted washing in combination with Thymus vulgaris essential oil nanocapsules. Sci. Rep..

